# PARP-1 Is a Potential Marker of Retinal Photooxidation and a Key Signal Regulator in Retinal Light Injury

**DOI:** 10.1155/2022/6881322

**Published:** 2022-09-10

**Authors:** Xun Li, ZiYuan Zhang, Bin Fan, YuLin Li, DeJuan Song, Guang-Yu Li

**Affiliations:** ^1^Jilin Provincial Institute of Education, Changchun 130022, China; ^2^Ophthalmology Department, Second Hospital of Jilin University, Changchun 130000, China

## Abstract

Advancements in technology have resulted in increasing concerns over the safety of eye exposure to light illumination, since prolonged exposure to intensive visible light, especially to short-wavelength light in the visible spectrum, can cause photochemical damage to the retina through a photooxidation-triggered cascade reaction. Poly(ADP-ribose) polymerase-1 (PARP-1) is the ribozyme responsible for repairing DNA damage. When damage to DNA occurs, including nicks and breaks, PARP-1 is rapidly activated, synthesizing a large amount of PAR and recruiting other nuclear factors to repair the damaged DNA. However, retinal photochemical damage may lead to the overactivation of PARP-1, triggering PARP-dependent cell death, including parthanatos, necroptosis, and autophagy. In this review, we retrieved targeted articles with the keywords such as “PARP-1,” “photoreceptor,” “retinal light damage,” and “photooxidation” from databases and summarized the molecular mechanisms involved in retinal photooxidation, PARP activation, and DNA repair to clarify the key regulatory role of PARP-1 in retinal light injury and to determine whether PARP-1 may be a potential marker in response to retinal photooxidation. The highly sensitive detection of PARP-1 activity may facilitate early evaluation of the effects of light on the retina, which will provide an evidentiary basis for the future assessment of the safety of light illumination from optoelectronic products and medical devices.

## 1. Introduction

The retina is responsible for sensing light signals from the outside world and converting these light signals into bioelectrical signals to form vision [1]. The frequency and duration of human eye exposure to excessive, strong, artificial lighting has gradually increased, including prolonged exposure to mobile phone screens, televisions, computers, excessive indoor illumination, and ophthalmic examinations with intensive lighting [2]. Although certain compensatory mechanisms exist in the retina to autorepair such damage, prolonged exposure to intensive light can cause acute or chronic retinal injury [3]. Therefore, how to effectively and accurately evaluate the safety of light illumination to the eye, especially to the retina, is still a necessary and urgent scientific issue.

Light-induced damage to the retina can be classified as photothermal damage and photochemical damage [4]. High-power light irradiation is able to cause a rapid increase in the temperature of local retinal tissue and can lead to irreversible retinal photothermal damage due to the protein denaturation and the inactivation of enzymes if the temperature rises more than 10°C above the basal level [5–8]. However, the light intensity that causes retinal photochemical damage is near the level of light illumination used in daily life; thus, retinal damage caused by photochemical reactions has become a growing concern [9]. Photooxidation is the initial molecular step that triggers the retinal photochemical damage via the oxidative cascade reactions and may even further activate the death signals in retinal cells [10]. *In vivo* studies have shown that retinal photochemical damage predominantly occurs in the outer layers of the retina, including the photoreceptor and retinal pigment epithelial (RPE) layers [11–13], as a large number of photosensitive substances, such as rhodopsin, melanin, and all-*trans*-retinal, are present in the cells located in these regions of the eye [14]. Energy from only an individual photon can be transferred to these photoactive groups, resulting in the modification of molecular structures and photooxidation [15].

Currently, the techniques most widely used to evaluate retinal light damage in vitro and in vivo assess three factors. First, retinal structural changes are determined using retinal slice hematoxylin and eosin (HE) staining, transparent electron microscope observation of ultrastructure, in vivo imaging optical coherence tomograms (SD-OCT), and autofluorescence (AF). Second, retinal functional changes are assessed via electroretinography, multifocal electroretinogram, visual field, or microvisual field. Third, the protein and mRNA levels of specific molecular markers retinal damage are measured. Light-induced changes in molecular levels may precede structural and functional changes, which may be an early indicator of the hazards of light illumination and retinal light damage and may be used a marker for early detection and assessment of these hazards [10]. Studies have shown that some markers, such as heme oxygenase 1 (HO-1) [16], 8-oxoG (related to oxidative stresse damage), caspase 3 [17], caspase 8 [18] (caspase-dependent apoptotic markers), as well as in situ terminal deoxynucleotidyl transferase dUTP nick-end labeling (TUNEL) [19], and rhodopsin levels, may indicate the severity of retinal light damage; however, whether these molecular markers are suitable for accurate and rapid assessment of retinal light damage requires further investigation.

Poly(ADP-ribose) (PAR) polymerase-1 (PARP-1) is a ribozyme that is involved in repairing DNA damage [20]. Studies have shown that PARP-1 is rapidly activated when DNA is damaged, and activated PARP-1 can synthesize a large amount of PAR to facilitate the recruitment of nuclear factors for DNA repair [21]. We and other teams have shown that excessive light radiation can lead to the significant upregulation of PARP-1 *in vivo* and *in vitro*, indicating that light radiation may cause nuclear DNA damage in retinal cells [22–24]. However, the overactivation of PARP-1 may lead to the exhaustion of cellular energy or trigger PARP-1-dependent death [25, 26]. Multiple lines of evidence support the notion that the inhibition of PARP-1 activity or knockdown of PARP-1 may play a key protective role against light damage in photoreceptor cells [27, 28]. Therefore, in this review, we summarize the molecular mechanisms of retinal photooxidation, PARP activation, and DNA repair and illustrate the connections among them. We determine the possibility of using PARP-1 as a standard marker for response to retinal light damage and elucidate the key regulatory role of PARP-1 in light-induced retinal injury. Highly sensitive and accurate detection of PARP-1 activity may facilitate the rapid assessment of the effects of light on the retina and evaluation of the safety of light illumination from various optoelectronic products and medical devices.

## 2. Methods

The MEDLINE, Scopus, and Wiley online databases were searched using multiple combinations of the keywords “PARP-1,” “photoreceptor,” “retinal light damage,” “photooxidation,” “parthanatos,” “necroptosis,” “autophagy,” and “mTOR.” Articles published between January 1, 1990 and January 1, 2022 were retrieved; articles without an available English translation were excluded.

## 3. ADP Ribosylation and PARP-1

ADP ribosylation is a reversible posttranslational modification (PTM) that covalently links one or more ADP ribose unit(s) to a target protein using *β*-nicotinamide adenine dinucleotide (*β*-NAD^+^) as a donor [29]. This modification is mediated by PARP family proteins, and the target protein can be modified by a single ADP ribose unit or by a polymer chain composed of multiple ADP ribose units [30]. The modification is classified as mono ADP ribosylation or poly ADP ribosylation, according to the amount of ADP ribose added to the target protein [31]. *In vitro* studies have shown that the modification of poly ADP ribosylation can contain up to 200 ADP ribosomes, including linear chains and branched chains [32]. ADP ribosylation can cause functional changes in the modified protein or can serve as a scaffold molecule to recruit other proteins to perform their functions after the protein is modified [33, 34]. Poly ADP ribosylation can regulate a variety of cellular processes, including cell division, apoptosis, chromatin structure regulation, transcription, and protein degradation [35].

The PARP family of proteins is responsible for catalyzing the ADP ribosylation modification [36]. The family consists of 17 members, and the catalytic domains of all members contain a classical conservative sequence H-Y-E, in which histidine and tyrosine are necessary for binding NAD^+^, while glutamic acid is related to catalytic activity [37]. According to the catalytic activity, the members can be classified into mono (ADP ribosyl) transferases (MARTS), poly(ADP ribosyl) transferases (PARTS), and inactive enzymes. MARTS include PARP-3, PARP-4, PARP-6, PARP-10, PARP-14, PARP-15, and PARP-16; PARTS include PARP-1, PARP-2, PARP-5a, and PARP-5b; and PARP-9 and PARP-13 do not have catalytic activity [38].

**Figure 1:**
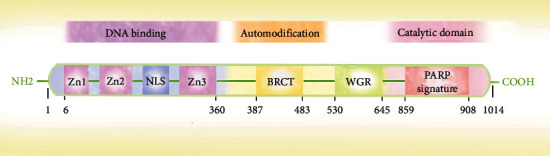
Three main domains of PARP-1 protein: (1) an N-terminal DNA binding domain, containing three zinc finger motifs (Zn1-3) and a nuclear localization sequence (NLS) that can identify DNA double- and single-stranded breaks; (2) a central BRCA1 C-terminal (BRCT), which is mainly for auto-modification; and (3) a C-terminal catalytic domain containing a tryptophan-glycine-arginine–rich domain (WGR) and PARP featured motifs, which is the binding site of the nicotinamide adenine dinucleotide (NAD) required for PAR synthesis.

PARP-1 was the first to be discovered, and it is also the most well-studied member of the PARP family. PARP-1 can uniquely catalyze ADPr residues to form long- branched chains of poly-ADPr polymers (PARylation) [20]. The human PARP-1 gene consists of 23 exons and is located on chromosome 1q42.12. The full-length PARP-1 protein is composed of 1,014 aa and has a molecular weight of approximately 116 kDa [39]. The structure of PARP-1 is composed of three functional domains: (1) an N-terminal DNA binding domain, containing three zinc finger motifs (Zn1-3) and a nuclear localization sequence (NLS), which can identify DNA double-stranded break and single-stranded breaks; (2) a central BRCA1 C-terminal (BRCT), which is an automodification domain that mediates protein-protein interactions; and (3) a C-terminal catalytic domain containing a tryptophan-glycine-arginine–rich domain (WGR) and PARP featured motifs, which is the NAD+ binding site required for PAR synthesis [40] (Figure 1). As a ribozyme, PARP-1 not only participates in DNA damage repair but is also involved in various biological functions, such as DNA replication, transcription regulation, cell cycle modulation, inflammation, differentiation, aging, and RNA processing [20].

## 4. Photooxidation and PARP-1 Activation

Prolonged exposure to intense visible light, especially exposure to the short-wavelength visible light with high energy, such as blue, violet, and green light, is prone to induce retinal photochemical damage [9]. The occurrence and severity of retinal photochemical damage is positively correlated with light energy intensity and exposure duration in a dose-dependent manner. Photooxidation is the initial step that triggers retinal photochemical damage [10]. As a crucial part of the visual system, the retina contains a large number of photosensitive groups for receiving light signals [41]. Cones contain three types of opsins that are sensitive to blue light (absorption peak at 430 nm), green light (absorption peak at 540 nm), and red light (absorption peak at 570 nm). All-*trans*-retinal in the outer segment of photoreceptor cells participates in the visual cycle with an absorption peak at 382 nm. In addition, A2E in RPE cells is one of the components of lipofuscin and may function as a potent photosensitizer with absorption peaks at 336 and 430–439 nm. Melanin is also present in RPE cells with an absorption peak at 335 nm [42]. The high energy carried by the photons of short-wavelength light in the visible light spectrum can trigger the orbital transition of electrons or break chemical bonds, resulting in modifications of molecular structures, once absorbed by the photosensitive groups of the retina [43]. The photon energy transferred to these photosensitive molecules causes the electron orbital transition of oxygen to generate singlet oxygen (1O^2^), which can react with other molecules to break their chemical bonds and further generate superoxide radicals (O^2·−^), hydrogen peroxide (H_2_O_2_), hydroxyl radicals (·OH), and other reactive oxygen species (ROS) [44]. This process is called photooxidation [45].

Excessive light irradiation can induce the production of a large amount of ROS in the retina. However, the imbalance between excessive accumulation of ROS and the ability of antioxidant defense systems to combat it may result in oxidative-stress damage, which can transduce the oxidative damage to cellular macromolecules, such as proteins, lipids, and DNA [46]. Notably, nitric oxide can penetrate the nuclear membrane and cause oxidative damage to DNA [47]. The bases, nucleotides, and single and double strands of nuclear DNA are all targets of ROS. Oxidative damage to DNA includes the modification of bases and the breaking of chemical bonds. Excessive ROS can even lead to structural modification of the four DNA bases (adenine, cytosine, guanine, and thymine). As these structures are modified, normal base pairing is disrupted. Oxidative damage to bases can cause base misincorporation, mismatches, and substitutions, ultimately leading to genetic mutations [48]. Guanine is the most easily oxidized DNA base because of its low oxidation potential, and the most common oxidized form is 8-oxo-2′-deoxyguanosine (8-oxoG) [49]. The major oxidized forms of the other three DNA bases are 8-oxo-2′-deoxyadenosine (8-oxoA, the oxidation product of adenine), thymidinediol (the oxidation product of thymine), and 5-hydroxy-2′-deoxycytidine (the oxidation product of cytosine) [50]. Oxidative damage can also break the hydrogen bonds between nucleotides, resulting in DNA single- or double-strand breaks or DNA gaps [51]. Free radicals then bind a hydrogen atom from the pentose of DNA to form a free radical with an unpaired electron at the C4 position, which in turn causes a break in the DNA chain at the *β*-position [52]. O^2^ can also decompose nucleotides, especially guanylate [53]. After oxidative damage, DNA may undergo fragmentation, mutation, and changes in thermal stability, which markedly affects gene transcription and translation [54].

**Figure 2:**
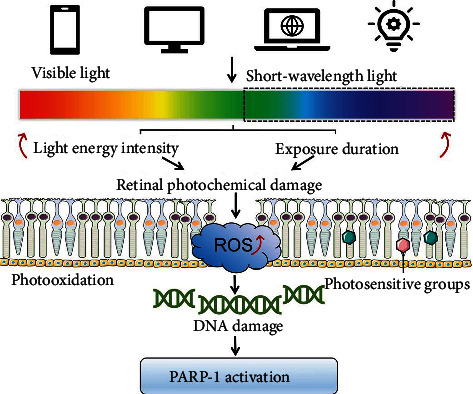
Correlation between PARP-1 activation and photooxidation. Prolonged exposure to intense visible light can induce retinal photochemical damage in a dose- and time-dependent manner. Photooxidation results in DNA damage in retinal cells; PARP-1 is then rapidly activated by the damaged DNA and participates in DNA repair. ROS: reactive oxygen species.

As a ribozyme, PARP-1 is responsible for repairing damaged DNA. The DNA damage such as DNA alkylation, strand gaps, and breaks can rapidly lead to the activation of PARP-1 [55, 56]. The catalytic activity of PARP-1 depends on its interaction with damaged DNA. As PARP-1 binds to DNA strand breaks, the PARP-1 activity increases and the resulting PAR synthesis is more than 500-fold higher than at basal levels [57]. The automodification of PARP-1 in response to DNA damage is crucial for rapid DNA repair and recruitment of nuclear factors to DNA injury sites [58–61]. In light-induced retinal damage, many studies have shown that PARP-1 responds rapidly and the expression level of PARP-1 increases significantly. Lara et al. found that exposure to white light (2200 lux, 24 hours) significantly increased the number of TUNEL-positive cells in the outer nuclear layer of the rat retina, accompanied by a significant increase in PARP-1 expression, while treatment with the antioxidant EGCG significantly mitigated this retinal light damage [62]. Moreover, our team showed that exposure to visible light markedly induced oxidative-stress damage in retinal ganglion cell 5 (RGC-5) cells *in vitro*, accompanied by a significant increase in the expression of PARP-1, and the PARP-1 inhibitor NU-1025 significantly protected RGC-5 cells from light damage [23]. Additionally, Liu et al. confirmed that visible light irradiation significantly induced excessive production of intracellular ROS, decreased the ratio of reduced/oxidized glutathione (GSH/GSSG), and overexpressed PARP-1 in cultured 661W cells *in vitro* [27]. Lv et al. demonstrated that exposure to light for 12 hours resulted in significant structural damage of the inner nuclear layer (INL) and ganglion cell layer (GCL) in mouse retinas and that this light exposure caused a significant upregulation of PARP-1 in a time-dependent manner [24]. Thus, these studies indicate that photooxidation may result in the damage of nuclear DNA in retinal cells, while the ribozyme PARP-1 may be rapidly activated by damaged DNA and participates in the repair of the DNA damage (Figure 2).

## 5. Role of PARP-1 in DNA Repair

Often, the damaged DNA sites are on only one strand, which are referred to as single-strand breaks (SSBs). There are many types of SSBs including breaks of the DNA backbone with intact base pairs, abasic sites caused by base (pyrimidine/purine) deletion, and nucleotide deletion. Since repair of SSBs can be guided by genetic information from the complementary strand, SSBs are usually easily mended [63]. However, a damaged site at the same position on both strands of DNA at the same time results in a double-strand break (DSB). DSBs are a more severe type of DNA damage and require the activation of special signaling pathways, such as the homologous recombination (HR) or nonhomologous end joining (NHEJ) [64].

**Figure 3:**
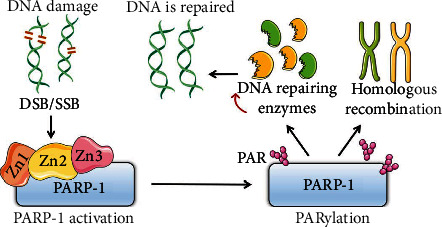
Role of PARP-1 in DNA repair response. PARP-1 can rapidly bind single-strand breaks (SSBs) and double-strand breaks (DSBs) with its zinc finger structures and trigger PARP-1 activation and PARylation, resulting in homologous recombination and the recruitment of DNA-repairing enzymes to repair the damaged DNA.

PARP-1 can rapidly identify DNA damage through zinc finger structures [65, 66]. Two of the single zinc fingers (Zn1 and Zn2) of human PARP-1 can form complexes with nucleotide bases exposed from on DNA double-strand gaps through a loop structure connecting with two *β*-strands [66]. In addition, zinc fingers can also identify the continuity of nucleotide bases and phosphate backbones through a “backbone grasping” mechanism [67]. Thus, PARP-1 is highly sensitive to DNA damage. Once identified DNA free ends caused by single- or double-stranded DNA gaps are sensed, PARP-1 can rapidly bind SSBs and DSBs with its N-terminal DNA binding domain. The binding of DNA gaps triggers a conformational transformation exposing the enzymatic site of PARP-1, resulting in PARP-1 activation and PARylation [56] (Figure 3). This PARylation response is very rapid; upon activation of PARP-1, intranuclear PAR levels can rise as much as 500-fold above baseline, consuming up to 90% of intracellular NAD^+^ [57]. Long or branched PARs on PARP-1 and other protein substrates serve as scaffolds for the recruitment of DNA repairing enzymes, facilitating the localization of repairing factors to DNA damaged sites [68]. For instance, X-ray repair cross-complementary protein 1 (XRCC1) is recruited by PAR chains and is a key scaffolding protein for the assembly and activation of DNA base excision repair machinery [69]. In the case of DSBs, the chromatin surrounding a break is rapidly and transiently PARylated to recruit the nucleosome remodeling and deacetylase (NuRD) complex, which can further result in ATP-dependent chromatin remodeling, histone deacetylation, and the recruitment of DNA repairing factors [70]. PAR at DSB lesions can also rapidly recruit meiotic recombination 11 (MRE11) to detect DSBs via HR or NHEJ, causing the halting of the cell cycle and the activation of downstream repair factors [70]. PARylation of chromatin-associated PARP-1 with negative charges causes chromatin decondensation and changes chromatin into an open conformation that facilitates DNA repair [71]. Furthermore, in alternative NHEJ pathways, PARylation caused by PARP-1 interacting with DSB ends leads to the recruitment of repairing proteins, DNA ligase III/XRCC1, and polynucleotide kinase phosphatase (PNKP) [72, 73]. PARylation modification at DNA damage sites and protein targets is highly dynamic, since PARylation can be rapidly degraded with half-lives ranging from 40 seconds to 6 minutes [74]. The degrading enzymes of PAR include poly(ADP-ribose) glycohydrolase (PARG), Nudix hydrolase 9 (NUDT9) and NUDT16, terminal ADP-ribose protein glycohydrolase 1 (TARG1), MacroD1, and MacroD2, which all contain the domain recognizing PAR and ADP-ribose [75–77]. PARG, as the major de-PARylation enzyme that localizes to the nucleus, can hydrolyze ribose-ribose bonds between ADP-ribose units [75], while NUDT9 and NUDT16 may hydrolyze phosphodiester bonds between ADP-ribose moieties and proteins [76, 77]. Removal of a terminal ADP-ribose is regarded as a rate-limiting step in PAR degradation, and TARG1, MacroD1, and MacroD2 may hydrolyze PARylation by cleaving a glutamate-banded ADP-ribose [75]. Accumulating evidence supports the important role of de-PARylation in DNA repair, however, the detailed mechanism of PARylation needs to be further elucidated.

When the retina suffers from photochemical damage, photooxidation is triggered and causes excessive accumulation of ROS within the retina, resulting in DNA damage of the outer layers of retinal cells. PARP-1 is then activated rapidly and promotes the repair of damaged DNA, to a certain extent, through PARylation. However, if the DNA damage is severe, PARP-1 will be overactivated, leading to cellular energy depletion and ultimately triggering PARP-1-dependent death.

## 6. PARP-1 Involvement in Signaling Pathways and PARP-Dependent Cell Death

### 6.1. Parthanatos

**Figure 4:**
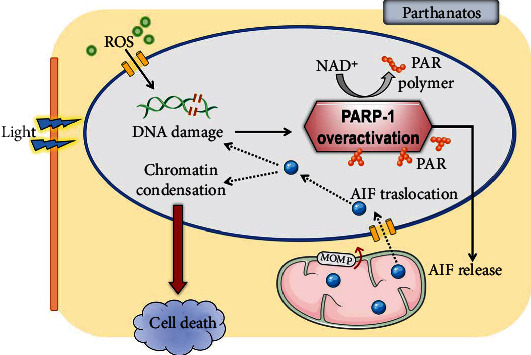
Parthanatos induced by light injury. Prolonged light exposure results in extensive DNA damage in the nucleus. PARP-1 is then overactivated leading to synthesis of a large amount of PAR and increase in mitochondrial outer membrane permeabilization (MOMP), triggering the release of apoptosis-inducing factor (AIF) from mitochondria, causing chromatin condensation/more DNA fragmentation and eventual cell death.

Parthanatos is a caspase-independent cell death characterized by the activation of PARP-1 and the nuclear translocation of apoptosis-inducing factor (AIF) [78]. Increasing evidence suggests that the parthanatos plays a crucial role in the progression of various neurodegenerative diseases [79, 80]. When death stimuli result in extensive DNA damage in the nucleus, PARP-1 is overactivated and synthesizes a large amount of PAR, which consumes massive intracellular NAD^+^ and ATP, eventually leading to energy exhaustion and cell death [81]. In addition, the intracellular accumulation of PAR may increase mitochondrial outer membrane permeabilization (MOMP), triggering the release of AIF from mitochondria [82]. AIF is a flavoprotein synthesized in the cytosol with a full-length precursor of 67 kDa (pre-AIF) [78]. The 67 kDa AIF is guided by its mitochondrial localization sequence (MLS) and translocates into the mitochondrial intermembrane space, where it is cleaved into the mature form 62 kDa AIF and participates in mitochondrial energy synthesis. Under death stimuli, 62 kDa AIF is hydrolyzed into the 57 kDa soluble form, tAIF, and released from the mitochondria into the cytosol. In the cytosol, tAIF can further translocate into the nucleus and interact with histone H2AX and endonucleases/DNases, causing chromatin condensation, large-scale DNA fragmentation, and cell death [83, 84] (Figure 4). Our recent study showed that exposure to visible light significantly induced the upregulated expression of PARP-1 in photoreceptor cells (661 W) *in vitro*, accompanied by the nuclear translocation of AIF, and knock down of PARP-1 with lentivirus-mediated shRNA significantly blocked nuclear translocation of AIF, thus protecting photoreceptor cells from light damage [22]. Lv et al. found that exposure to light for 12 h resulted in significant structural damage of the inner nuclear layer (INL) and ganglion cell layer (GCL) of the mouse retina and that light irradiation significantly increased the level of PARP-1 in a time-dependent manner. In addition, they found that light exposure also caused the activation of the PARP-1/AIF signaling pathway in RGC-5 cells (retinal precursor neurons) cultured *in vitro* and that the PARP inhibitor NU1025 significantly attenuated the light-induced death of RGC-5 cells [24].

### 6.2. Necroptosis

Necroptosis, a form of programmed cell death, is initiated by tumor necrosis factor-*α* (TNF-*α*) signaling and mediated by receptor-interacting protein kinase 1 (RIPK1) and RIPK3 [85], though it is caspase-independent [86] and can be blocked by necrostatin-1 (Nec-1) [87]. Studies have shown that PARP-1 is involved in TNF-*α*-induced necroptosis. PARP-1 can regulate necroptosis directly by interacting with RIP kinases or indirectly by generating PAR, which in turn can target necroptosic effectors [88]. The activity of PARP-1 is also influenced by upstream RIP kinases, and its activity is significantly increased during TNF-*α*-induced necroptosis, while inhibition of PARP-1 blocks necroptosis, suggesting that PARP-1 plays an active role in necroptosis [89]. Xu et al. showed that the activation of PARP-1 was involved in glutamate-induced necroptosis in HT-22 cells, and necrostatin-1, an inhibitor of necroptosis, could reduce the activity of PARP-1 [90]. Hitomi et al. demonstrated that PARP-2 played a key role in necroptosis in L929 cells induced by TNF-*α* and a caspase inhibitor (zVAD-fmk), while knockout of PARP-2 significantly inhibited necroptosis [91]. However, the *in vitro* evidence from Sosna et al. suggested that TNF-*α*-induced necroptosis and PARP-1 signaling represent two distinct and independent programmed necroptosis pathways [92]. Although studies have shown that PARP-1 is closely related to the necroptosis pathway, the exact molecular mechanism of this interaction remains to be elucidated. In addition, there is a lack of *in vivo* experimental evidence determining whether PARP-1 is directly or indirectly involved in necroptosis regulation, particularly clarifying the specific crosstalk between PARP-1 and TNF-*α* or RIP.

### 6.3. Autophagy

**Figure 5:**
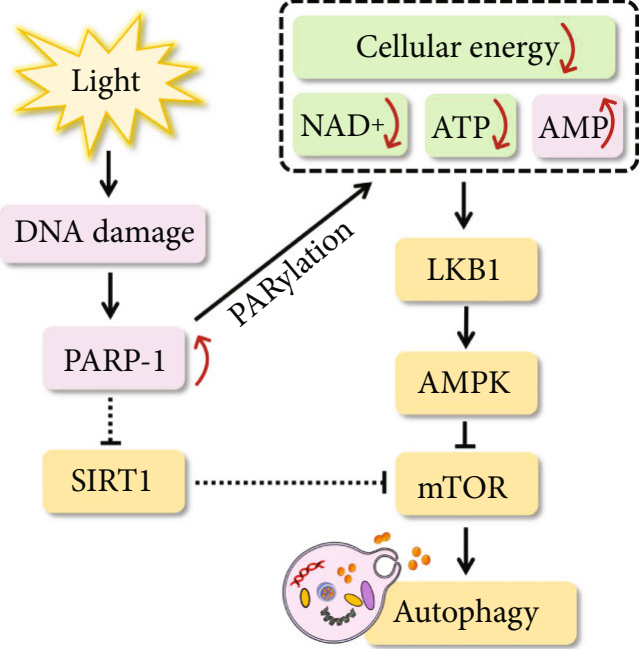
PARP-1-dependent autophagy. The massive synthesis of PAR (PARylation) by the overactivation of PARP-1 leads to NAD^+^ and ATP depletion and significant increases in intracellular AMP, which remarkably activates autophagy through the interaction between the AMP-activated protein kinase (AMPK) and mammalian target of rapamycin (mTOR) signaling pathways. In addition, sirtuin 1 (SIRT1) may be the signal hub between PARP-1 and the mTOR signaling pathway.

Autophagy is a programmed self-degradation process used to maintain cellular energy homeostasis. By degrading damaged or dysfunctional organelles, cells may recycle amino acids, lipids, and other molecules via autophagy [93]. The activation of autophagy is closely related to intracellular energy status and is regulated by the mammalian target of rapamycin (mTOR)/AMP-activated protein kinase (AMPK) signaling pathway [94]. For example, mTOR may negatively regulate the activation of autophagy. Mammalian target of rapamycin (mTOR) remains active under conditions where there is sufficient nutrient supply to keep autophagy “off,” while mTOR is significantly inhibited as cells are starved, which in turn results in the activation of autophagy to promote energy production [95]. AMPK is able to sense the changes of cellular ATP/AMP and regulate autophagy activation through its downstream signal, mTOR [96]. The massive synthesis of PAR by the overactivation of PARP-1 leads to NAD^+^ and ATP depletion as well as significant increases in intracellular AMP, which remarkably influence the activation of autophagy through the interaction between the AMPK/mTOR signaling pathways [97] (Figure 5). Chen et al. showed that PARP-1 promotes autophagy in CNE-2 cells after ionizing radiation by activating AMPK and inhibiting mTOR [98]. Huang et al. showed that, in addition to causing ATP consumption, the overactivation of PARP-1 also promotes autophagy through the liver kinase B1 (LKB1)-AMPK-mTOR pathway, thereby enhancing cell survival in oxidative stress-induced DNA damage [99]. Excessive light exposure can not only cause an increase in the level of PARP-1 but also in the activation of mTOR in photoreceptors [22, 100]. Pan et al. demonstrated that PARP-1 knockdown reduced the phosphorylation level of mTOR in photoreceptors *in vitro*, while knockdown of mTOR also resulted in a significant decrease in the levels of PARP-1 and PAR and that sirtuin 1 (SIRT1) may be the signal hub between PARP-1 and the mTOR signaling pathway [22]. However, as the underlying modulator of the interaction between mTOR and PARP-1, the role of SIRT-1 in regulating photodamage-induced autophagy still needs to be further elucidated.

## 7. Conclusions and Perspectives

**Figure 6:**
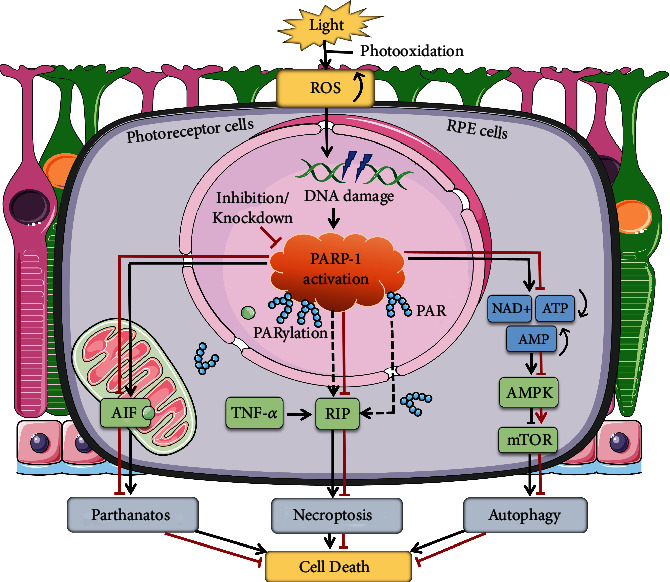
Schematic diagram illustrating that PARP-1 may be a key signal regulator in retinal light injury. Prolonged exposure to intense visible light may cause retinal photochemical damage that is triggered by photooxidation. Oxidative stress damage can cause nuclear DNA breaks and lead to rapid and significant activation of PARP-1 to repair damaged DNA. Additionally, inhibition or knockdown of PARP-1 can play a crucial protective role against light damage in the outer layers of retinal cells.

Prolonged exposure to intense visible light, especially short-wavelength light such as blue light, may cause retinal photochemical damage, which is predominantly caused by intracellular cascade reactions triggered by photooxidation [9]. Intracellular oxidative stress damage can cause DNA breaks in the nucleus, in turn triggering the activation of the ribozyme, PARP-1, for DNA repair [99]. Therefore, determination of PARP-1 levels may indirectly reflect retinal light damage. Since PARP-1 is highly sensitive to DNA damage, it can be rapidly activated and is significantly upregulated once DNA damage occurs [101], ultimately triggering PARP-1-dependent cell death (Figure 6). PARP-1 has better stability and sensitivity compared with other oxidative stress markers for evaluating retinal light damage, such as HO-1 or 8-oxoG. Experimental evidence for assessing the safety of light illumination on the retina may be provided by determining the level of PARP-1 after light irradiation, using *in vitro* and *in vivo* experimental models and may also be indicative of light illumination safety in daily work.

The detection of PARP-1 activity is crucial as PARP-1 may serve as a potential biomarker. Frequently used techniques for detecting PARP-1 include enzyme-linked immunosorbent assay (ELISA), biotin labeling, immunoblotting, fluorescence, and colorimetry [102–106]. Recently, novel methods have been developed for more efficient detection of PARP-1 activity. Liu et al. indicated a potential tool for PARP-1 activity detection based on the large impact of PARP-1 on the diffusion flux of ferricyanide in anodic aluminum oxide (AAO) nanochannels [107]. Zhou et al. proposed a method to linearly detect PARP-1 activity based on host–guest recognition using a renewable electrochemical (EC) sensor modified with mono-(6-mercapto-6-deoxy)-beta-cyclodextrin on the electrode surface to avoid unspecific adsorption and improve detection accuracy [108]. Liu et al. developed an ultrasensitive EC detection for PARP-1 activity on basis of the electrostatic interaction of PAR and polyaniline [109]. Wang et al. designed a label-free photoelectrochemical (PEC) biosensor, also based on the electrostatic interaction of PAR and another chemical compound, poly [9,9-bis(6′-N, N, N-trimethylammonium) hexyl] fluorenylene phenylene for detection of PARP-1 activity [110]. In addition, Xu et al. further developed a dual-mode (both EC and PEC), label-free strategy for the detection of PARP-1 activity through gold nanocluster (AuNCs). The AuNCs adsorbed by PAR produced both strong fluorescence and chemiluminescence by catalyzing the luminol-H_2_O_2_ system, which provided higher sensitivity and stability, a wider linear range, and better biocompatibility [111]. In conclusion, these highly sensitive methods for detecting PARP-1 activity may facilitate rapid assessment of retinal light damage, providing an evidentiary basis for future evaluation of the safety of light illumination produced by optoelectronic products and medical devices.
